# A Trend Analysis of Adherence to the Muscle Strengthening Exercise Guidelines in US Adolescents

**DOI:** 10.3389/ijph.2022.1605022

**Published:** 2022-11-15

**Authors:** Sitong Chen, Jin Yan, Yaping Zhao

**Affiliations:** ^1^ Institute for Health and Sport, Victoria University, Melbourne, VIC, Australia; ^2^ Centre for Active Living and Learning, University of Newcastle, Callaghan, NSW, Australia; ^3^ College of Human and Social Futures, University of Newcastle, Callaghan, NSW, Australia; ^4^ The Library Unit, Shandong Sport University, Jinan, Shandong, China

**Keywords:** secular changes, muscle activity, the Youth Risk Behavior Surveillance, adolescent, disparity

## Abstract

**Objectives:** This study aimed to describe the trends of the muscle-strengthening exercise (MSE) guidelines adherence in adolescents and factors associated with the adherence.

**Methods:** Using the Youth Risk Behavioural Survey data, this study assessed the trends of adhering to the MSE guidelines in adolescents. The survey-year-based trends of MSE guidelines adherence was assessed with logistic regression. Binary logistic regression was used to identify the correlates (i.e., sex, grade, race/ethnicity) of the guidelines’ adherence.

**Results:** 73,074 study participants (female = 36,497, male = 36,108; mean age = 16.04 years) were included for analysis. An overall declining trend of the MSE guidelines in adolescents was found (55.6% in 2011 → 49.5% in 2019, *p* < 0.001), and similar trends were observed in both sexes. The declining trends varied by sociodemographic factors (e.g., grade). Boys and younger adolescents were more likely to adhere to the MSE guidelines.

**Conclusion:** The declining trend of adhering to MSE guidelines in US adolescents would be a health concern in this population. Girls and older adolescents should be targeted as intervention priorities.

## Introduction

Strong epidemiological research evidence demonstrates that muscle-strengthening exercise (MSE) is an independent factor of various health outcomes, such as all-cause mortality [1, 2], diabetes [3, 4] and psychological health [5]. As a branch of physical activity epidemiology research on MSE currently receives public health importance and research interest because of the convincing evidence. Generally, MSE refer to strength/weight/resistance training activity or exercise, which is a voluntary activity that includes the use of weight machines, exercise bands, hand-held weights, or own body weight (e.g., push-ups or sit-ups) [6]. Owing to the health benefits, the current evidence-based guidelines recommend that adults should do MSE at least two times a week while children and adolescents should do three or more times a week [6].

Theoretically, the recommendations on MSE provide a benchmark for surveillance and monitoring on MSE at the population level [6] and some epidemiological survey studies shed lights on the secular trends of adhering to the MSE guidelines as investigations tracking changes in MSE guidelines adherence can respond to relevant behavioural interventions. For example, some studies used the national data to describe the changes of adhering to the MSE guidelines in American adults [7–10], and gaining mixed results. Despite the inconsistent trends, these emerging studies based on adults can at least map the current trend patterns and in turn inform feasible health initiatives to promote MSE of adults.

Beyond the efforts of surveillance and monitoring on trends in MSE in adults, less is studied on people under 18 years, even though children and adolescents can benefit from sufficient MSE [11, 12]. To date, a very few studies described the trends in MSE in adolescents. For instance, a study based on Canadian youth found a linear decrease in adherence to the MSE guidelines [13]. Moreover, adolescence is a key window period that adolescents can develop healthy lifestyle behaviours and then maintain it into later life. It is needed to conduct surveillance and monitoring of the specific healthy behaviours during adolescence [14]. Identifying how specific trend patterns of MSE vary according to population characteristics can assist in understanding adolescent subgroups’ behaviour changes, which is then helpful to inform specific intervention approaches. In addition to knowing the trends pattern, conducting research on correlates of MSE guidelines’ adherence is another approach to identify who are at-risk of insufficient MSE participation. This information also assists in identifying adolescents’ subgroups who need prioritised intervention aimed at increasing MSE. However, such evidence remains very rare in adolescents.

The primary aims of this study, therefore, was to (1) describe secular trends of adhering to MSE guidelines using a nationally representative sample of US adolescents between 2011 and 2019; (2) describe how trends vary by sociodemographic factors (sex, grade and race/ethnicity); (3) examine sociodemographic factors of adhering to the MSE guidelines based on the Youth Risk Behaviour Surveillance (YRBS) data.

## Methods

### Study Design

This study used data from five cycles of the YRBS (2011, 2013, 2015, 2017, 2019). The YRBS is a biennial, cross-sectional school-based survey of health-related behaviours among a nationally representative sample of high school students living in the United States. More details on the YRBS can be found through the link (https://www.cdc.gov/healthyyouth/data/yrbs/index.htm). With such data, a repeated cross-sectional study was conducted to observe trends of adherence to the MSE guidelines in adolescents. All data included for our study was approved, which is ethically suitable for trend analysis.

### Participants

The YRBS uses a three-stage cluster sample design to recruit students attending public and private schools in grades 9 to 12 (mean age: 14.7 years of 9 grade; 15.6 years of 10 grade; 16.6 years of 11 grade; 17.5 years of 12 grade). The survey was administered in person by trained data collectors and completed by students during class time. Overall response rates were above 60% during the administration of each cycle of the YRBS. The survey results were weighted to represent the populational and national health data. The data used in this secondary analysis were deidentified and publicly available; hence, no protocol approval from an institutional review board was needed. Additional details about the YRBS can be found by accessing the website (https://www.cdc.gov/healthyyouth/data/yrbs/index.htm). Prior to data collection, study participants were informed survey purposes and they were voluntarily to participate in the YRBS survey for protecting their own privacy. Based on this, the pooled data in our study was ethnically allowed.

### Measures

#### Sociodemographic

Participants provided sociodemographic information pertaining to their sex (female/male), grade (9, 10, 11, 12), and race/ethnicity (White, Black/African American, Hispanic/Latino, Other).

#### Adherence to MSE According to the World Health Organization

Using a paper-pencil questionnaire, participants were asked “During the past 7 days, on how many days did you do exercises to strengthen or tone your muscles, such as push-ups, sit-ups, or weightlifting?”. The answer options were 0–7 days. Participants reporting 3 or more days were considered to adhere to the MSE guidelines according to the WHO [15]. This measure has been confirmed to have psychometric properties in adolescents in different countries [13, 16–18], and all the measures in the YRBS are reliable and valid [19].

### Statistical Analysis

All the variables included in this study were treated as categorical variables. For each variable, weighted prevalence estimates with 95% confidence intervals (CIs) were calculated while accounting for complex sampling surveys using Taylor linearization to produce nationally representative prevalence estimates for each survey year. To examine trends in the prevalence of adhering to the MSE guidelines across the 2011 to 2019 rounds of the YRBS, logistic regression models were conducted with time-trend variables that assessed secular changes across the years of data. Survey year was used as a continuous variable to assess the linear trend, and quadratic terms of survey year were included to examine the quadratic trend. Only linear time variable was included in the logistic regression models to examine the linear trends, both linear and quadratic time variables were included when examining the quadratic trends [20, 21]. Separate logistic regression models were also performed to explore associations between sociodemographic variables (sex, grade, race/ethnicity) and MSE guideline adherence, which generated a year-specific association. Adjusted odds ratios (ORs) with 95% confidence intervals (95% CIs) after controlling for sex, grade, and race/ethnicity are presented for all logistic regression models. All analyses were performed using survey prefix command (SVY; this command was designed for complex survey data) procedures taking sampling stratum, primary sampling unit and weight based on the YRBS protocol in Stata/IC 17.0 Basic Edition (Stata Corp LLC). Statistical significance was considered at a 2-tailed *p*-value less than 0.05.

## Results

The unweighted and weighted sample characteristics for the overall sample and each survey year’s sample are presented in Table 1. Of all 73,074 samples, the proportion of males and females was almost equal (49.9% female; 49.4% male; 0.6% missing). Similarly, the proportion of the sample of 9–12th grades was almost equal. In terms of race/ethnicity, the White sample predominantly accounted for over 40%. Information on the sample of each survey year is presented in detail in Table 1, and more information on the weighted sample characteristics can be found as well.

**TABLE 1:** Sample characteristics of this study (the Youth Risk Behaviour Surveillance, 2011, 2013, 2015, 2017 and 2019, the United States).

	Total		2011		2013		2015		2017		2019	
	Unweighted	Weighted	Unweighted	Weighted	Unweighted	Weighted	Unweighted	Weighted	Unweighted	Weighted	Unweighted	Weighted
	N (%)	% [95% CI]	N (%)	% [95% CI]	N (%)	% [95% CI]	N (%)	% [95% CI]	N (%)	% [95% CI]	N (%)	% [95% CI]
Sex												
Girl	36,497 (49.9)	49.4 [48.4–50.4]	7,708 (50.0)	48.4 [46.7–50.2]	6,621 (48.7)	50.0 [48.7–51.2]	7,757 (49.6)	48.7 [45.5–51.9]	7,526 (51)	50.7 [48.2–53.2]	6,885 (50.3)	49.4 [48.0–50.8]
Boy	36,108 (49.4)	50.6 [49.6–51.6]	7,656 (49.6)	51.6 [49.8–53.3]	6,950 (51.2)	50.0 [48.8–51.3]	7,749 (49.6)	51.3 [48.1–54.5]	7,112 (48.2)	49.3 [46.8–51.8]	6,641 (48.6)	50.6 [49.2–52.0]
Missing	469 (0.6)		61 (0.4)		12 (0.1)1		118 (0.8)		127 (0.9)		151 (1.1)	
Grade												
9	18,923 (25.9)	27.3 [26.5–28.0]	3,774 (24.5)	27.7 [25.8–29.7]	3,588 (26.4)	27.3 [26.0–28.7]	4,003 (25.6)	27.2 [25.4–29.1]	3,921 (26.6)	27.3 [26.0–28.7]	3,637 (26.6)	26.6 [25.3–28.0]
10	18,215 (24.9)	25.7 [25.0–26.3]	3,693 (23.9)	25.8 [24.1–27.5]	3,152 (23.2)	25.7 [24.7–26.7]	3,938 (25.2)	25.7 [23.8–27.7]	3,715 (25.2)	25.7 [24.8–26.6]	3,717 (27.2)	25.5 [24.7–26.4]
11	18,171 (24.9)	24.0 [23.5–24.4]	4,133 (26.8)	23.9 [22.9–24.9]	3,184 (23.4)	23.9 [22.9–24.9]	3,930 (25.2)	24.0 [22.7–25.2]	3,602 (24.4)	23.9 [23.2–24.6]	3,322 (24.3)	24.3 [23.3–25.3]
12	17,090 (23.4)	23.1 [22.6–23.6]	3,699 (24.0)	22.6 [21.4–23.9]	3,557 (26.2)	23.1 [21.9–24.3]	3,601 (23)	23.1 [22.2–24.0]	3,383 (22.9)	23.1 [22.1–24]	2,850 (20.8)	23.6 [22.4–24.8]
Missing	675 (0.9)		126 (0.8)		102 (0.8)		152 (1)		144 (1)		151 (1.1)	
Race/ethnicity												
White	31,398 (43)	54.4 [51.7–57.1]	6,171 (40)	56.9 [49.3–64.1]	5,449 (40.1)	55.6 [47.4–63.6]	6,849 (43.8)	54.5 [46.7–62.1]	6,261 (42.4)	53.5 [46.9–59.9]	6,668 (48.8)	51.2 [43.9–58.4]
Black or African American	12,263 (16.8)	13.6 [12.2–15.0]	2,767 (17.9)	14.2 [10.7–18.5]	2,993 (22)	14.3 [10.1–20.1]	1,667 (10.7)	13.6 [10.2–17.9]	2,796 (18.9)	13.4 [10.5–17.1]	2040 (14.9)	12.2 [9.0–16.3]
Hispanic/Latino	19,828 (27.1)	22.4 [20.4–24.6]	4,627 (30.0)	20.0 [15.0–26.2]	3,395 (25)	21.1 [16.3–27.0]	5,121 (32.8)	22.3 [16.6–29.2]	3,647 (24.7)	22.8 [17.7–28.9]	3,038 (22.2)	26.1 [20.1–33.2]
All other races	7,819 (10.7)	9.7 [8.7–10.7]	1,545 (10.0)	9.0 [7.2–11.2]	1,428 (10.5)	8.9 [7.2–11.0]	1,629 (10.4)	9.7 [7.7–12.0]	1724 (11.7)	10.3 [8.8–12.0]	1,493 (10.9)	10.5 [7.9–13.8]
Missing	1766 (2.4)		315 (2.0)		318 (2.3)		358 (2.3)		337 (2.3)		438 (3.2)	

**FIGURE 1:**
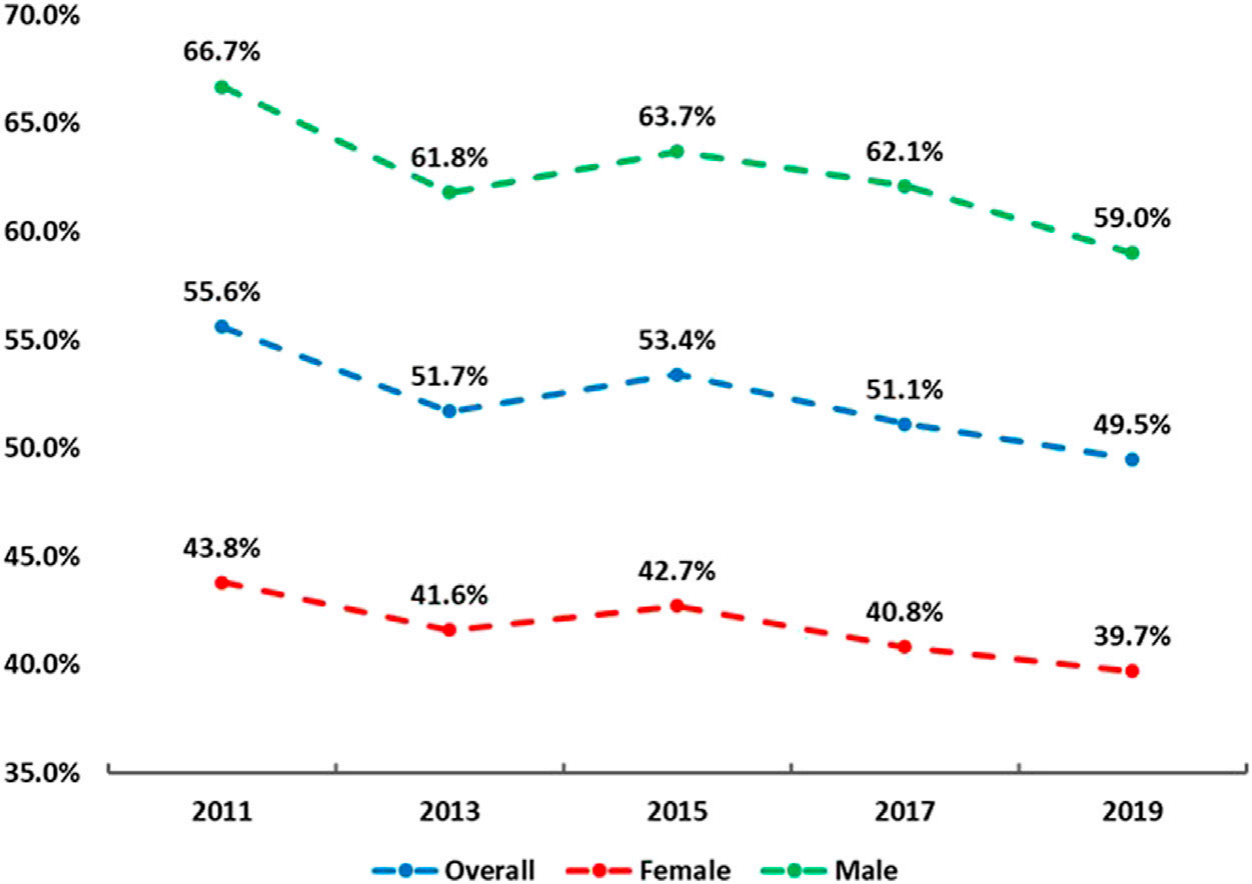
Trends in adherence to the muscle strengthening exercise guidelines in adolescents by overall and sex (the Youth Risk Behaviour Surveillance, 2011, 2013, 2015, 2017 and 2019, the United States).

**FIGURE 2:**
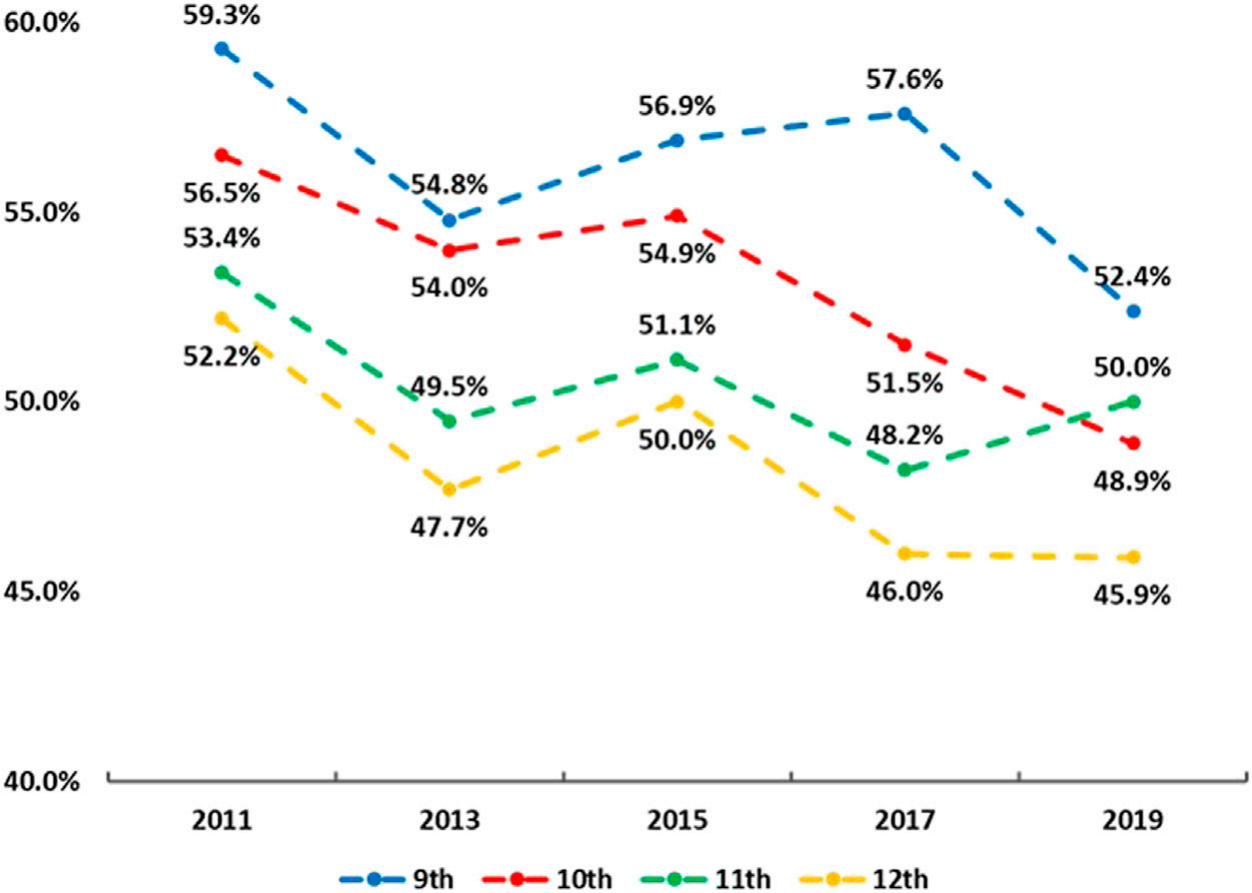
Trends in adherence to the muscle strengthening exercise guidelines in adolescents by grades (the Youth Risk Behaviour Surveillance, 2011, 2013, 2015, 2017 and 2019, the United States).

**FIGURE 3:**
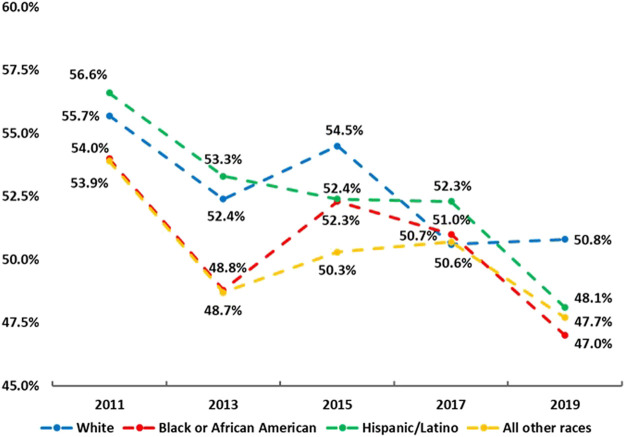
Trends in adherence to the muscle strengthening exercise guidelines in adolescents by race/ethnicity (the Youth Risk Behaviour Surveillance, 2011, 2013, 2015, 2017 and 2019, the United States).

Figure 1 shows trends of adhering to the MSE guidelines in the sample from 2011 to 2019. In the overall sample, a linear declining trend of adhering to the MSE guidelines was found (55.6% in 2011 → 49.5% in 2019; OR = 0.98, 95% CI: 0.96–0.99, *p* < 0.001). A similar linear trend was also found in both boys (66.7% in 2011 → 59.0% in 2019; OR = 0.97, 95% CI: 0.95–0.98, *p* < 0.001) and girls (43.8% in 2011 → 39.7% in 2019; OR = 0.98, 95% CI: 0.96–0.99, *p* = 0.044). Figure 2 depicts the trends of adhering to the MSE guidelines in the sample by grades from 2011 to 2019. In sample of grade 9, declining patterns were observed in 2011 (59.3%) and 2013 (54.8%), and 2017 (57.6%) and 2019 (52.4%), with an increase (∼2%) between 2013 and 2015. In sample of grade 10, from 2011 to 2019 (except between 2013 and 2015, a slight increase), consistent declining patterns were found, resulting in a 6.5% decrease from 2011 to 2019. This in general resulted in a non-significant linear trend (*p* = 0.067). Similarly, in the sample of grade 11, a non-significant linear trend was found (*p* = 0.130). Conversely, in the sample of grade 10 and 12, a linearly declining trend was detected respectively (both *p* < 0.05). For example, the prevalence of adhering to the MSE guidelines in sample of grade 10 decreased from 56.5% in 2011 to 50.0% in 2019 (OR = 0.97, 95% CI: 0.95–0.98, *p* < 0.001). Figure 3 displays the trends of adhering to the MSE guidelines in the sample by race/ethnicity. It was observed that sample in all other races did not have significant trend from 2011 to 2019 (*p* = 0.265). In the sample of White, Black or African and Hispanic/Latino, linear declining trends were also found. For example, in the sample of Hispanic/Latino, the prevalence of adhering to the MSE guidelines decreased from 56.6% in 2011 to 48.1% in 2019 (OR = 0.97, 95% CI: 0.94–0.99, *p* < 0.005). More information about parameters on the trends can be found at Table 2.

**TABLE 2:** Trends for meeting the muscle strengthening exercise guidelines (the Youth Risk Behaviour Surveillance, 2011, 2013, 2015, 2017 and 2019, the United States).

	Trends	Meeting the MSE guidelines			*p* value
		OR	95% CI		
Overall	Linear	0.98	0.96	0.99	0.000
	Quadratic	1.00	0.99	1.01	0.917
Boy	Linear	0.97	0.95	0.98	0.000
	Quadratic	1.00	0.99	1.01	0.997
Girl	Linear	0.98	0.96	0.99	0.044
	Quadratic	1.00	0.99	1.01	0.867
9th	Linear	0.98	0.96	1.00	0.067
	Quadratic	1.00	0.99	1.01	0.665
10th	Linear	0.97	0.95	0.98	0.000
	Quadratic	1.00	0.99	1.00	0.309
11th	Linear	0.98	0.96	1.01	0.130
	Quadratic	1.01	1.00	1.01	0.299
12th	Linear	0.97	0.96	0.99	0.010
	Quadratic	1.00	0.99	1.01	0.862
White	Linear	0.98	0.96	0.99	0.019
	Quadratic	1.00	0.99	1.01	0.980
Black or African American	Linear	0.97	0.94	0.99	0.023
	Quadratic	1.00	0.99	1.01	0.683
Hispanic/Latino	Linear	0.97	0.94	0.99	0.004
	Quadratic	1.00	0.99	1.01	0.877
All other races	Linear	0.98	0.96	1.01	0.265
	Quadratic	1.00	0.99	1.02	0.686

MSE, muscle strengthening exercise; OR, odd ratio; CI, confidence interval.

Table 3 presents the results for the association between some selected sociodemographic factors and adherence to the MSE guidelines. In the overall sample, males were more likely to meet the MSE guidelines (OR = 2.36, 95% CI: 2.23–2.29) than their counterparts. Such a result was also found in the sample of each survey year. For grades, it was generally found that compared with 12 graders, the younger sample was more likely to meet the MSE guidelines. Interestingly, in the overall samples, taking the sample of Black or African Americans, only the White sample had a greater likelihood (OR = 1.11, 95% CI: 1.02–1.21) of adhering to the MSE guidelines.

**TABLE 3:** Association between sociodemographic factors and adherence to the muscle strengthening exercise guidelines (the Youth Risk Behaviour Surveillance, 2011, 2013, 2015, 2017 and 2019, the United States).

	Total			2011			2013			2015			2017			2019		
	OR	95% CI		OR	95% CI		OR	95% CI		OR	95% CI		OR	95% CI		OR	95% CI	
Male	2.36	2.23	2.49	2.55	2.32	2.81	2.31	2.07	2.58	2.37	2.11	2.66	2.37	2.02	2.77	2.18	1.96	2.42
Female	REF																	
9th grade	1.40	1.30	1.51	1.38	1.17	1.63	1.35	1.15	1.59	1.32	1.12	1.55	1.65	1.39	1.95	1.31	1.13	1.51
10th grade	1.24	1.14	1.34	1.22	1.04	1.43	1.31	1.07	1.61	1.25	1.05	1.49	1.25	1.07	1.47	1.14	0.98	1.33
11th grade	1.10	1.03	1.17	1.07	0.93	1.23	1.09	0.96	1.24	1.06	0.89	1.25	1.09	0.96	1.23	1.19	1.02	1.39
12th grade	REF																	
White	1.11	1.02	1.21	1.06	0.89	1.27	1.14	0.99	1.32	1.12	0.89	1.41	1.03	0.84	1.27	1.23	0.99	1.51
Hispanic/Lantin	1.06	0.97	1.17	1.08	0.86	1.35	1.19	1.01	1.40	1.01	0.82	1.24	1.05	0.85	1.29	1.11	0.89	1.39
All other races	0.99	0.90	1.09	0.97	0.79	1.21	0.99	0.86	1.15	0.92	0.70	1.19	1.03	0.87	1.21	1.11	0.86	1.42
Black or African American	REF																	

OR, odds ratio; CI, confidence interval; REF, reference group for comparison with other subgroups.

## Discussion

The current study primarily aimed to describe the trends of adhering to the MSE guidelines using a large nationally representative sample in the US and then explore the association between the selected sociodemographic factors and adhering to the MSE guidelines. It was mainly found that the US adolescents experienced a declining trend of adhering to the MSE guidelines from 2011 to 2019. We also found that males and adolescents with lower grades had higher odds of adhering to the MSE guidelines, while race/ethnicity may not be a significant correlate of MSE in adolescents. Detailed discussions are further presented below.

The results of the current study suggested in general, a declining trend of adhering to the MSE guidelines in US adolescents. Bennie et al. [13] found that the prevalence of adhering to the MSE guidelines declined significantly from 2015 to 2019 using nationally representative data of Canadian youth. Our results were consistent with Bennie et al.‘s [13] study, which can partly explain the decreased muscular fitness in youth [11, 22, 23]. Of note, our data based on the YRBS allowed us to perform a longer trend than showed Bennie et al. [13], and we analysed the trends by sociodemographic factors, which advances knowledge in the literature. Additionally, in our study, declining trends were differently found in adolescents across grades and of different races/ethnicities. For some specific subgroups of adolescents, it seems impossible to gain insightful understanding of why these subgroups of adolescents did not show significantly linear trends, as we failed to know the information on the data collection procedure and sampling bias. However, we can initially confirm that the trends of adhering to the MSE guidelines may be influenced by sociodemographic factors. Future studies are urged to explore why the different trends occur, which can in turn better help some subgroups of adolescents’ MSE for early interventions.

Concerning the reasons for the declining trends in MSE in US adolescents, to date, no studies can provide sensible and well-discussed explanations. Indeed, approximately half of the US adolescents failed to meet the MSE guidelines according to our study results. This prevalence is lower than that in Chinese youth [16] but consistent with Canadian youth [13, 24]. Much evidence has suggested that participating in MSE is associated with various health benefits [12, 25–27], so the declining trends observed in the current study are worrisome. As the declining trends may persist into later life, immediate and effective interventions should be implemented. However, unlike research on physical activity intervention, improving MSE is still an understudied research topic in the literature [6, 28]. In the future, an increasing number of studies should fill these research gaps.

Identifying the correlates of MSE in youth is of great importance to MSE interventions and improvement for health promotions. In terms of sociodemographic correlates of adhering to the MSE guidelines, the current study found that males were more likely to meet the MSE guidelines than females. This research finding can be supported by many previous studies [13, 17, 29]. It is possible that females have more barriers, including insufficient time and a lack of exercise skills, to engage in MSE than males [16, 30]. Our study was also consistent with early research showing age-related declines in MSE in adolescents [16, 17, 29]. Interestingly, when aggregating all the survey data to explore the association between race/ethnicity and adhering to the MSE guidelines, we found that compared with Black or African American adolescents, only White adolescents were more likely to meet the MSE guidelines. However, when examining the data by each survey year, the above noted research finding disappeared, which we can presume that this inconsistency would be attributed to sample size. Although the current study explored the association between sociodemographic factors and MSE, these correlates are unmodifiable. However, based on these research findings, intervention-prioritized subgroups in young people could be determined. Future studies are expected to determine correlates of MSE at more dimensions, such as interpersonal, environmental, or cultural aspects.

Owing to the standardised measurement protocol of the YRBS, we should point out some methodological considerations to researchers for better understanding of our research findings. The most important research matter involves the measurement of MSE. In our study, assessing MSE was based on one single question, which only focused on frequency of MSE per week owing to the standardised measurement protocol of the YRBS. It is recently recognised that assessing MSE at the population level should consider more components, such as duration, type and intensity [31, 32]. Such data, however, is lacking in our study but it is urgently needed to address this research issue for better capturing information on MSE in the future. Similar to the research issue on assessment of MSE, evidence concerning correlates of MSE in our study is also limited as only a few numbers of potential factors were assessed in the YRBS measurement protocol. Exploring correlates of adhering to the MSE guidelines in children and adolescents has received sufficient research attention and priority [16, 18], but evidence remains scanty. However, in our study, only some sociodemographic factors were included, which may inhibit research insights into broadly and multidimensionally understanding MSE behaviour in children and adolescents. These two research considerations are recommended to improve future research.

This study is not without limitations. On the one hand, the self-reported measure used in our study may be subject to study participants’ recall memory and social desirability. On the other hand, when exploring the correlates of MSE, owing to cross-sectional data, it cannot draw any conclusive evidence. To better understand our research findings, cautions should be taken when treating these study limitations. Some study strengths worth mentioning include the use of a standardized survey process and nationally representative sample recruitment, which can enhance the generalizability of the research findings.

### Conclusion

As one of very first to assess the trends in adherence to the muscle strengthening exercise guidelines in adolescents using a nationally representative sample, this trend analysis suggested that the US adolescents experienced a declining trend in adherence to the muscle strengthening exercise. From the behavioural perspective, female and older adolescents should be targeted as intervention priorities to enhance their engagement in muscle strengthening exercise, which in turn promotes public health at the populational level. When designing the specific interventions, sex- and age- related differences should be taken into consideration. The research findings of this study can help design and implement efficient muscle strengthening exercise interventions in adolescents.
